# Fusion of CTA and CTB gene to immunogen significantly enhances the immunogenicity of DNA vaccine

**DOI:** 10.1186/1742-4690-9-S2-P8

**Published:** 2012-09-13

**Authors:** X Ren, Y Wan, X Xie, J Xu

**Affiliations:** 1 Fudan University, Shanghai, China; 2 SuZhou University, SuZhou, China

## Background

Cholera toxin and its two subunits (CTA,CTB) have been intensively investigated as mucosal adjuvants for protein based vaccine. In this study we evaluated the adjuvanticity of CTA and CTB in modality of either mixing their encoding plasmids with DNA vaccine or fusing their encoding genes to immunogen encoding gene.

## Methods

DNA and recombinant vaccinia vaccines expressing HIV-1 AE strain tat, rev, intergrase(C-half), vif, nef fusion gene(designated as TRIVN) have been constructed. For the construction of fusion gene of CTA/CTB to TRIVN, overlapping PCR was employed to link CTA/CTB and TRIVN gene, the fused genes(TRIVN-CTA and TRIVN-CTB)were cloned into eukaryotic expression plasmid vector(pSV1.0). Six groups of female BALB/c mice were immunized with mock control, pSV-TRIVN, pSV-TRIVN-CTA, pSV-TRIVN-CTB, pSV-TRIVN mixed with CTA or with CTB respectively in a DNA priming-recombinant vaccinia boosting regimen. Two weeks after the final injection, mice splenocytes were collected and IFN-γ ELISPOT assay were used as readout for specific T cell response. Statistical analysis was performed by using Prism5.0 software.

## Results

Our data showed that all constructed plasmids are capable of efficiently expressing their inserted genes. All groups immunized with vaccines raised significant more T-cell response than mock control. T-cell responses elicited by pSV-TRIVN-CTB(1548±330SFCs/106splenocytes) and pSV-TRIVN-CTA (1642±514SFCs/106splenocytes) were significantly higher than that by pSV1.0-TRIVN (520±150SFCs/106splenocytes), pSV1.0-TRIVN mixed with CTA (692±220SFCs/106splenocytes) and pSV1.0-TRIVN mixed with CTB (734±240SFCs/106splenocytes). Though TRIVN-CTA and TRIVN-CTB fusion vaccines mounted comparable level of total IFN-γ+ T-cell responses, only TRIVN-CTB elicits significantly T-cell responses against Tat, which is a subdominant component in the fusion immunogen. No significant differences were observed among groups inoculated with TRIVN alone or adjuvanted by CTA/CTB subunit proteins.

## Conclusion

CTA and CTB could serve as potent adjuvants for DNA vaccine in immunogen-CTA/CTB fusion modality. Compared with CTA, CTB may enhance T-cell responses against subdominant epitopes in the immunogen and broaden the T-cell immune responses.

